# Social and Physical Environments and Disparities in Risk for Cardiovascular Disease: The Healthy Environments Partnership Conceptual Model

**DOI:** 10.1289/ehp.7913

**Published:** 2005-07-18

**Authors:** Amy J. Schulz, Srimathi Kannan, J. Timothy Dvonch, Barbara A. Israel, Alex Allen, Sherman A. James, James S. House, James Lepkowski

**Affiliations:** 1 Health Behavior and Health Education, and; 2 Environmental Health Sciences, School of Public Health, University of Michigan, Ann Arbor, Michigan, USA; 3 ISLES, Inc., Trenton, New Jersey USA; 4 Duke University, Durham, North Carolina, USA; 5 Survey Research Center and Department of Sociology, and; 6 Institute for Social Research and Department of Biostatistics, University of Michigan, Michigan, Ann Arbor, USA

**Keywords:** community-based participatory research partnerships, racial segregation and cardiovascular disease, social and physical environments and cardiovascular disease

## Abstract

The Healthy Environments Partnership (HEP) is a community-based participatory research effort investigating variations in cardiovascular disease risk, and the contributions of social and physical environments to those variations, among non-Hispanic black, non-Hispanic white, and Hispanic residents in three areas of Detroit, Michigan. Initiated in October 2000 as a part of the National Institute of Environmental Health Sciences’ Health Disparities Initiative, HEP is affiliated with the Detroit Community–Academic Urban Research Center. The study is guided by a conceptual model that considers race-based residential segregation and associated concentrations of poverty and wealth to be fundamental factors influencing multiple, more proximate predictors of cardiovascular risk. Within this model, physical and social environments are identified as intermediate factors that mediate relationships between fundamental factors and more proximate factors such as physical activity and dietary practices that ultimately influence anthropomorphic and physiologic indicators of cardiovascular risk. The study design and data collection methods were jointly developed and implemented by a research team based in community-based organizations, health service organizations, and academic institutions. These efforts include collecting and analyzing airborne particulate matter over a 3-year period; census and administrative data; neighborhood observation checklist data to assess aspects of the physical and social environment; household survey data including information on perceived stressors, access to social support, and health-related behaviors; and anthropometric, biomarker, and self-report data as indicators of cardiovascular health. Through these collaborative efforts, HEP seeks to contribute to an understanding of factors that contribute to racial and socioeconomic health inequities, and develop a foundation for efforts to eliminate these disparities in Detroit.

Cardiovascular disease (CVD) is the largest contributor to all-cause mortality in the United States and accounts for one-third of the excess mortality experienced by non-Hispanic black compared with non-Hispanic white Americans (Wong et al. 2002). Although CVD risk has declined substantially over the past 30 years, this decline has been uneven across both socioeconomic position (SEP) and racial or ethnic groups, resulting in increasing disparities (Cooper et al. 2000; Williams 1999). Although socioeconomic disparities and racial disparities in health risks and health outcomes between non-Hispanic black and non-Hispanic white Americans have been well established (Cooper et al. 2000; Cubbin et al. 2001; Diez-Roux et al. 1999; Kaplan and Keil 1993; Lynch et al. 1996, 1997; Wong et al. 2002), mixed results are reported in the literature on CVD risk and mortality among Mexican Americans (Hunt et al. 2003; Luepker 2001; Pandey et al. 2001; Sorlie et al. 1993; Sundquist and Winkleby 2000; Winkleby et al. 1999).

Understanding the patterns and processes associated with racial disparities in CVD is an important priority for health professionals, and perhaps more so for the communities that disproportionately experience CVD morbidity and mortality. The Healthy Environments Partnership (HEP) is a community-based participatory research (CBPR) partnership that brings together representatives from community-based organizations, public health organizations, and academic institutions to examine the contributions of social and physical environmental contexts to the risk of CVD. In this article we describe the conceptual model that guides HEP’s work, the study design, and the processes used to facilitate engagement among these diverse partners in the development and implementation of this study.

## Background

### Cardiovascular disease in Detroit.

Residents of Detroit experience age-adjusted risks of death due to heart disease that are considerably higher than either the Michigan or the national rates (Table 1). CVD mortality rates for non-Hispanic black Detroiters were not substantially higher than for non-Hispanic blacks in Michigan or nationally (relative risk, 1.1 and 1.2, respectively), but mortality rates among non-Hispanic white Detroit residents were substantially higher than either the Michigan or the national rates (relative risk, 1.5–1.6, respectively). Although data were not available for Detroit’s predominantly Mexican American Hispanic population, the literature for Mexican Americans elsewhere in the United States is mixed: some report lower risk of CVD (Mitchell et al. 1990) or mortality (Sorlie et al. 1993), whereas others report similar or higher rates of CVD mortality among Mexican Americans compared with non-Hispanic whites (Hunt et al. 2003; Luepker 2001; Pandey et al. 2001). Sundquist and Winkleby (2000), reporting on a national sample of Mexican American women and men from the Third National Health and Nutrition Examination Survey (NHANES III), note the heterogeneity of the Mexican American population and suggest the importance of examining variations in both individual characteristics and contextual factors in understanding variations in cardiovascular risk.

CVD mortality rates also vary within Detroit. The 3-year age-adjusted average CVD mortality rate (2000–2002) on Detroit’s east side was 523.9; in northwest Detroit, 395.3; and in southwest Detroit, 426.9 (Michigan Department of Community Health 2003). Understanding the factors that account for these variations requires understanding contemporary and historical relationships between the city and the surrounding region, and within the city itself.

A thriving and prosperous community with a strong blue-collar middle class for much of the twentieth century, like many similar urban areas, Detroit experienced population out-migration and economic disinvestment beginning in the 1950s and escalating in the 1970s and 1980s. As Detroit’s population declined, surrounding suburban areas experienced unprecedented economic and population growth. These economic and population shifts were fueled by white fears of racial integration and the departure of most of the city’s white residents to suburban neighborhoods as African American residents moved into previously all-white Detroit neighborhoods (Sugrue 1996). The racial composition of Detroit shifted from 16% African American in 1950 to 83% in 2000 (Schulz et al. 2002). For the past two decades, Detroit has been among the most racially segregated metropolitan areas in the United States (Glaeser and Vigdor 2001; Sugrue 1996). Concurrently, employment opportunities relocated to outlying areas, contributing to an exponential growth in areas of concentrated poverty within the city.

House and Williams (2000) have noted that SEP shapes “people’s experience of and exposure to virtually all psychosocial and environmental risk factors for health. . . . [T]hese in turn operate through a very broad range of physiological mechanisms to influence the incidence and course of virtually all causes of disease and death” (p 83). SEP, whether measured by education, income, occupation, or a composite measure aggregating two or more of these indicators, is predictive of mortality across a wide range of health outcomes, including, but not limited to, CVD (House 2002). The pervasiveness of these influences has led some to suggest that SEP is a “fundamental factor” influencing health by shaping access to multiple resources needed to maintain health and avoid disease (Link and Phelan 1995). More recently, Williams and Collins (2001) have extended this argument, suggesting that race-based residential segregation is a fundamental factor influencing health disparities shaping differential access to multiple resources—including but not limited to, education, income, and wealth—necessary to maintain health. The HEP project focuses on explicating the effects of race-based residential segregation in concentrating access to political, economic, and social resources and the resulting implications for health.

### The Detroit HEP.

HEP was initiated in October 2000 as a part of the National Institute of Environmental Health Sciences Health Disparities Initiative and is affiliated with the Detroit Community–Academic Urban Research Center (URC) (Israel et al. 2001). The URC board, composed of representatives from community-based organizations, health service and public health institutions, and academic institutions, identified the contribution of environmental factors to health disparities as a priority. HEP contributes to this goal by examining aspects of the social and physical environments and their association with health status across areas within Detroit and by disseminating results from these analyses within the study communities as well as peer-reviewed venues.

HEP investigates the prevalence of biologic indicators of CVD and the extent to which these inequalities are mediated through social and physical environmental exposures, with implications for proximate factors such as health-related behaviors, psychosocial stressors and responses, and social integration. In addition, HEP aims to disseminate and translate findings to inform new and established intervention and policy efforts through HEP’s community outreach and education program (COEP).

HEP engages researchers based in academic institutions and representatives from health service organizations and community-based organizations in a collaborative effort to address these questions (see acknowledgments footnote on page 1 of this article for a list of HEP partner organizations). Representatives from the partner organizations comprise the HEP steering committee (SC), which is involved, in varying degrees, in all aspects of the research process. In 2001 the SC adopted a set of CBPR principles that emphasizes involving community, practitioner, and academic partners in all major phases of the research process; strengthening collaboration among all partners; conducting research that is beneficial to the communities involved; enhancing the capacity of all partners; and disseminating findings to community members in ways that are understandable and useful (Israel et al. 1998, 2005).

## The Healthy Environments Partnership Conceptual Model

**Figure 1:**
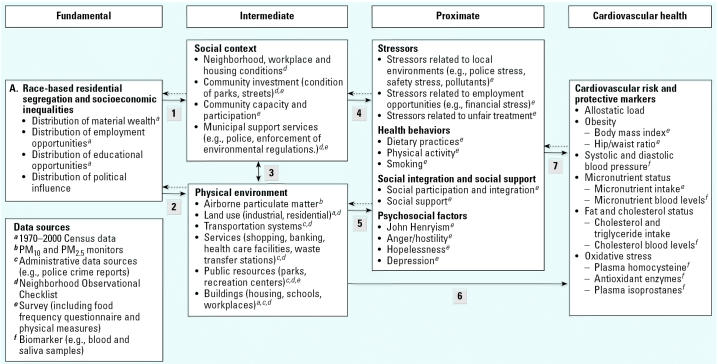
Conceptual model and data sources for HEP: social and physical environmental factors and disparities in cardiovascular risk. Arrows 1–7 indicate relationships between components of the conceptual model. Solid arrows indicate the main hypothesized effect. Dashed arrows indicate that some reciprocal effect may be present. Letters in the box “Data sources” refer to footnotes in other boxes in the figure.

The conceptual model that guides HEP’s work builds on previous CBPR efforts undertaken by the URC (Israel et al. 2001, 2002; Parker et al. 2001; Schulz et al. 2001); the literature describing relationships between SEP, racial segregation, and access to resources necessary to maintain health (House and Williams 2000; Link and Phelan 1995; Schulz et al. 2002; Schulz and Northridge 2004; Williams and Collins 2001); and the extensive literature on CVD. The HEP conceptual model shown in Figure 1 posits that the social and physical environments serve to mediate relationships between racial and socioeconomic inequalities (expressed in patterns of race-based residential segregation and concentrated poverty) and more proximate social, psychological, behavioral, and biologic indicators of CVD risk.

### Fundamental Factors: Race-Based Residential Segregation and Concentrated Poverty

Race-based residential segregation and economic inequality appear on the left of Figure 1 as fundamental factors influencing intermediate and proximate risks for CVD. Racial or ethnic status remains a major determinant of SEP in the United States as a result of interpersonal and institutional discrimination that constrains housing, educational, and employment opportunities (Conley 2000; House and Williams 2000). Similarly, there are steep gradients in risk for CVD mortality by SEP, whether measured as income, education, or occupation at the individual level (Cooper 2001; Kaplan and Keil 1993; National Heart, Lung, and Blood Institute 1995) or by indicators of income inequality (Cooper R, Casper M, Barnett E, unpublished data). The evidence linking race-based residential segregation to income inequality, as well as to constrained educational and economic opportunities within many predominantly black residentially segregated urban communities (Massey and Denton 1993; Orfield 1993), suggests mechanisms through which race-based residential segregation may contribute to CVD risk. At least one study (Cooper 2001) found an effect of race-based residential segregation on cardiovascular mortality above and beyond the effect of income inequality. HEP’s conceptual model posits that race-based residential segregation and associated economic inequalities influence the social and physical environments in which people live (Figure 1, arrows 1 and 2).

### Intermediate Factors: Social and Physical Environments

Our model conceptualizes social environments as social, economic, and political relationships at the local level, for example, workplace conditions, citizen engagement and influence, indicators of community investment, and municipal supports such as street maintenance and the capacity and cultural competence of the police force. The physical environment includes the built environment, such as age and quality of housing stock, transportation systems, and age and location of industrial activities, which in turn influence residents’ exposures to, for example, airborne pollutants.

To illustrate the concepts represented by arrows 1 and 2 in Figure 1, processes that concentrate poverty in racially segregated communities affect both household income and area tax bases (Farley et al. 2000; Wacquant and Wilson 1989; Wilson 1996). The availability of personal and municipal economic resources in turn influences the infrastructure that supports community life, such as the adequacy and competence of the police force, fire-fighting services, and other municipal supports (Sugrue 1996; Wacquant and Wilson 1989). Race-based residential segregation influences the distribution of educational and employment opportunities (Massey and Denton 1993; Orfield 1993, 2001); services and retail outlets (Sugrue 1996); health care providers and pharmacies (McLafferty 1982; Whiteis 1992); and parks and recreational facilities, grocery stores, and fast food and liquor establishments (LaVeist and Wallace 2000; Zenk et al. 2005a).

Differential access to economic resources also has implications for residents’ ability to influence local political decisions. Areas with high concentrations of poverty contain fewer individuals with the economic resources and political influence to shape decisions regarding, for example, land use or the enforcement of existing environmental regulations (arrow 3). Concentrating residents with few political and economic resources into specific areas of the city weakens political influence (Cohen and Dawson 1993) and contributes to increased risk of exposure to hazards in the physical environment (Maantay 2001). Among these is exposure to airborne particulate matter (PM), which is linked to increased risk of CVD (Pope et al. 2004; Samet et al. 2000; Verrier et al. 2002).

Effects of airborne PM on CVD have been demonstrated at levels below the U.S. National Ambient Air Quality Standards (Peters et al. 2001). Detroit residents experience considerable fluctuations in air quality, and all of metropolitan Detroit has been designated as a nonattainment area for PM ≤2.5 μm in aero-dynamic diameter (PM_2.5_) as of 2004. Recent measurements also suggest that residents of some areas within Detroit may be disproportionately exposed to elevated levels of respirable particles (Keeler et al. 2002). This may affect cardiovascular risk factors (arrow 6). In addition, aspects of the built environment and airborne PM may also influence cardiovascular risk indirectly, through more proximate factors such as physical activity, social integration and social supports, and exposure to chronic stressors (arrow 5).

### Proximate Factors and Cardiovascular Risk

Environmental conditions may influence a variety of more proximate risk factors, including perceived stressors, health-related behaviors, social integration and support, and psychosocial responses to stressors (arrows 4 and 5). Established variations in these risk factors by racial status and SEP may arise, at least in part, through the effects of the social and physical environments, exposure to stressful life conditions, health-related behaviors, social integration, and social support. Although a comprehensive review of this literature is beyond the scope of this article, we highlight established relationships between several proximate factors and CVD.

#### Stressful life conditions.

Exposure to stressful life events varies by SEP and race or ethnicity (Bosma et al. 1997; Marmot et al. 1997; Schulz et al. 2001; Williams et al. 1997), and the HEP conceptual model suggests that these variations are, at least in part, shaped by aspects of the social and physical environment. For example, residents of areas with few employment opportunities may experience higher levels of stressors related to job insecurity or inflexibility, or financial insecurity (Heslop et al. 2002; Pickering 1999; Wilson 1996). Similarly, in communities in which the tax base is inadequate to support police, firefighting, and other city services, residents may experience heightened concerns about crime, police effectiveness, and safety (Morenoff and Sampson 1997; Schulz and Lempert 2004).

Laboratory research on allostatic load, the body’s response to chronically stressful life conditions, has established that these physiologic responses experienced over time can lead to altered functioning of the hypothalamic–pituitary–adrenal axis and to increased risk of CVD (Björntorp 2001; Esch et al. 2002; McEwen 2000; Vitaliano et al. 2002). Excess cortisol produced under chronically stressful circumstances contributes to central adiposity (deposits of fat in the midsection of the body), an established risk factor for CVD (Björntorp 2001). Chronic exposure to stressful life conditions is linked to primary hypertension (Björntorp 2001) and may contribute to chronic inflammatory processes culminating in atherosclerosis (Black and Garbutt 2002).

#### Health-related behaviors.

Differences in health-related behaviors by race, ethnicity, and SEP may be influenced by differences in local social and physical environments (Lantz et al. 1998; Lynch et al. 1997; Zhang and Wang 2004). For example, both household income and residence in areas of concentrated poverty are associated with reduced intake of micronutrients that are protective against CVD (Kaufman et al. 1997). Residents of areas with high concentrations of poverty often experience reduced access to essential nutritional resources (Laraia et al. 2004; Nestle and Jacobson 2000; Swinburn et al. 1999; Zenk et al. 2005a). Intake of some micronutrients, including vitamins B_6_ and B_12_, which are cofactors in the metabolism of homocysteine, may interact with exposure to airborne PM to influence oxidative stress, a risk factor for CVD (Ford et al. 2002).

Inverse relationships have also been established between social class and smoking and may reflect in part a response to stressful life conditions associated with economic hardship (James 1999). Physical activity, another protective factor against CVD, may be influenced by conditions in the physical and social environment (Brownson et al. 2001; Lantz et al. 1998; Swinburn et al. 1999). Crespo et al. (1996), using NHANES data, found that 40% of African American women, who are disproportionately likely to live in communities with poorly maintained sidewalks and to have reduced access to recreational facilities, reported no leisure-time physical activity. Furthermore, a study of Latina women in an urban area found that concerns about safety were an impediment to outdoor physical activity (Kieffer et al. 2002).

#### Social integration and social support.

Social network ties, support, and integration vary in relation to SEP and are strongly associated with premature death and disease (Cacioppo et al. 2002; Heaney and Israel 1997), including CVD (Berkman et al. 1992; Case et al. 1992; Kawachi et al. 1996). The availability of social support when faced with stressful life conditions is also associated with depression and psychological distress (Israel et al. 2002; Lepore 1997; McEwen and Seeman 1999). There is some evidence that chronically stressful life conditions can contribute to erosion of these protective social relationships (Barrera 2000; Green and Rodgers 2001).

#### Psychosocial indicators.

Finally, a number of psychosocial characteristics have also been associated with increased risk of CVD, including anger or hostility (Carroll et al. 1997), and John Henryism, a high-effort coping response to stressful life conditions, with patterns that appear to be sensitive to social context (Dressler et al. 1998; James and Thomas 2000). Important health outcomes in their own right, symptoms of depression and psychological distress have also been found to be associated with cardiovascular mortality (Sheps and Sheffield 2001; Stansfield et al. 2002).

### Cardiovascular Risk and Protective Markers

The proximate risk factors described in the preceding discussion have been linked to physiologic indicators for CVD (arrow 7). These include blood pressure, body mass, hip:waist ratio, and hemostatic (e.g., cholesterol) indicators of cardiovascular risk. There is substantial evidence that these cardiovascular risks are differentially distributed by race, ethnicity, and SEP. Rates of hypertension and cardiovascular mortality (Mensah et al. 2005), abdominal obesity (Sundquist and Winkleby 2000), and diabetes (Harris et al. 1998) vary by race, ethnicity, and socioeconomic indicators.

In sum, Figure 1 describes pathways through which established racial and socioeconomic differences in CVD risk may be shaped by race-based residential segregation and income inequalities, mediated through social and physical environments. This conceptual model has guided the HEP’s efforts to examine independent and cumulative contributions of aspects of the environment to patterns of CVD in Detroit. In the remainder of this article we describe the HEP study design.

## Study Design

### Data Collection

The HEP study design was initially developed through discussion among members of the URC board before submission of the grant proposal. The URC had previously worked in two of the areas of the city included in this study; the board recommended adding the third (northwest Detroit) after discussing the research questions, to increase variation across study communities in air quality. The URC board helped to develop the HEP study design, and once funding was received, board members identified several new organizations from areas of the city involved in the study to join the HEP SC. In keeping with the principles of CBPR, members of the HEP SC worked together to design specific components of the study. As we describe each of the areas of the study below, we also describe briefly how members of the partnership worked together to design, implement, and interpret results from the study. [For additional details on the participatory processes involved, see Schulz et al. (2005a) and Zenk et al. (2005a).]

HEP used a wide range of data collection methods to address the study questions. These included data from decennial censuses (1970–2000; U.S. Census Bureau 2005); administrative sources (e.g., land use documents); neighborhood observation checklist (NOC); airborne PM ≤10 μm in aerodynamic diameter (PM_10_) and PM_2.5_ monitored in each of three study communities over a 3-year period (January 2000 through December 2002); a stratified random-sample community survey administered to residents of the three study communities; and biomarker data collected from a subset of survey participants. Approval was granted for the HEP study in January 2001 by the University of Michigan Institutional Review Board for Projection of Human Subjects.

#### Census and administrative data.

Data from the 1990 decennial census (U.S. Census Bureau 2005) were used to identify the three HEP study areas, based on evidence of variations in racial/ethnic and socioeconomic composition, as well as preliminary air quality data indicating variations in airborne PM. During the study period, a doctoral research assistant worked with the HEP SC to identify additional census data of interest and to compile data relevant to the study questions (e.g., percentage below/above poverty; median home value), for decennial censuses conducted between 1970 and 2000 (U.S. Census Bureau 2005). A postdoctoral scholar worked with the SC to identify sources of relevant administrative data (e.g., crime reports, location of parks and recreational facilities, toxic waste sites).

#### Neighborhood observation checklist.

A subcommittee of the HEP SC developed a systematic NOC to document characteristics of selected blocks within the areas from which survey respondents were sampled (see survey sampling description in “Community survey”). This subcommittee worked with a doctoral research assistant to adapt items from several existing instruments (Caughy et al. 2001; Farquhar 2000; Morenoff JD, House JS, Raudenush SW, unpublished data; Perkins et al. 1992) and to develop new items for this checklist through an extensive process (for a more complete description of this process, see Zenk et al. 2005b). The final 140-item checklist assessed aspects of the social and built environments for each study block (e.g., condition of homes and businesses, vacant lots, streets and sidewalks, traffic patterns, and parks and recreational facilities). Neighborhood raters completed a 36-hr initial training period followed by group and individual practice sessions, and feedback of interrater reliability (IRR) statistics based on practice blocks. Eleven observers were certified and collected data using the HEP NOC on 551 blocks across the three study neighborhoods during a 15-week period in the summer and early fall of 2003 (Zenk SN, Schulz AJ, Mentz G, House JS, Miranda P, Gravlee CC, et al., unpublished data; Zenk et al. 2005b). The sample for the NOC consisted of 147 blocks in which one or more HEP survey respondents resided, and 404 blocks that shared a common border with those blocks (so-called rook neighbors) (Lee and Wong 2001).

#### Physical environment: airborne particulate matter.

PM_10_ and PM_2.5_ were measured seasonally over a 3-year period (January 2000 through December 2002) as indicators of the physical environment in the three study communities. Data collected included a historical assessment of exposure to ambient PM_10_, as well as a multiyear assessment of exposure to fine aerosols, PM_2.5_, and the chemical components of PM_2.5_. This multiyear approach allowed proper characterization of community level exposure to PM_10_ and PM_2.5_ and attention to the contribution of point or localized sources of ambient air pollution (e.g., motor vehicle traffic, industrial facilities).

PM_2.5_ and PM_10_ samples were collected daily onto 47-mm Teflon membrane filters (Pall Life Sciences, Ann Arbor, MI) during seasonal measurement intensives [four times per year, 2 weeks duration each; see Keeler et al. (2002) for additional detail] using the dichotomous sequential air sampler Partisol-Plus (model 2025; Rupprecht and Patashnick Co., Inc., East Greenbush, NY), for subsequent chemical and elemental characterization of fine and coarse particles as previously described (Keeler et al. 2002). The dichotomous configuration of the sampler permits the differentiated mass determination and chemical composition of the fine (≤2.5 μm aerodynamic diameter) and coarse (2.5–10 μm) particles contained in PM_10_, which can aid in further source identification. Consistent with other aspects of the project, HEP SC members were involved as members of analysis and writing teams examining and disseminating the PM results.

#### Community survey.

The HEP community survey was developed by a survey subcommittee of the SC that worked together for over a year to develop and pretest the survey instrument. In doing so, this subcommittee drew on results from community focus groups, the literature on cardiovascular risk and protective factors, and extensive discussions between April 2001 and April 2002 (Schulz et al. 2005a). Survey data collection began March 2002 and ended March 2003.

The HEP survey sample is a stratified, two-stage equal probability sample of occupied housing units (or households) in the three areas of Detroit in which air quality was monitored (see “Physical environment: airborne particulate matter”). In each of the three areas, all respondents lived in a compact area with at most a 1.3-mile radius. The sample was designed to obtain 1,000 completed interviews with persons 25 or more years of age in the three study areas.

In each area, households were to be selected to attain approximately equal representation across racial and ethnic groups and by SEP. This design was intended to allow for comparisons across racial and ethnic and socioeconomic status while holding air quality constant (i.e., within geographic areas). It also allows comparisons of residents with similar social and economic characteristics across air quality exposures (i.e., across geographic areas of the city). The racial and ethnic distributions of the Detroit population did not allow study goals to be met completely. White residents were oversampled in northwest and southwest Detroit, and census tracts in southwest Detroit where most of the Hispanic population resides were oversampled. No effort was made to select Hispanic respondents from the two study areas in which Hispanic residents made up < 1% of the population, or white respondents in the east side of the city where there were fewer than 3% white residents.

In the first stage of selection, blocks were selected with probabilities proportionate to Census 2000 (U.S. Census Bureau 2000) counts of households. Households within sample blocks were listed by study staff, and a sample of approximately equal numbers of housing units per block were selected with probabilities inversely proportionate to size. The products of the probabilities of selection were equal for housing units in each study area.

Interviewers visited each sampled housing unit to complete the last stage of selection. They attempted to obtain a list of all residents 25 or more years of age. Respondents were randomly selected from the list of eligible household members using an objective respondent selection procedure (Kish 1965). Probabilities of selection were varied to achieve target numbers of non-Hispanic black, non-Hispanic white, and Hispanic participants of low and moderate socioeconomic status.

Study enrollment projections and results of field sampling are shown in Table 2. In east-side Detroit, which was 97% non-Hispanic black according to Census 2000 (U.S. Census Bureau 2000), 97% of HEP survey respondents reported their race as African American. In northwest Detroit, white respondents were oversampled, and interviews were completed with 162 non-Hispanic black and 93 non-Hispanic white respondents as well as 13 respondents with other or unspecified racial or ethnic identity. In southwest Detroit, both non-Hispanic white and Hispanic respondents were oversampled, and interviews completed with 93 non-Hispanic black, 99 non-Hispanic white, and 177 Hispanic respondents, most of whom identified as of Mexican origin. The number of survey participants with household incomes above and below the poverty line by race and area of the city are also shown in Table 2 compared with enrollment targets. Interviews were conducted in Spanish or in English according to the preference of the respondents: 106 interviews were completed in Spanish.

Of the 2,517 housing units in the initial sample, 1,297 were invalid (e.g., vacant, under construction), were unable to be screened after repeated attempts (no one contacted after 12+ attempts, refused screener), or contained no eligible respondent (e.g., no one 25 or more years of age). Of the 1,220 households in which an eligible respondent was identified, interviewers were unable to contact the identified respondent after repeated attempts in 193 (16%). Of the 1,027 eligible respondents contacted, 105 (10%) refused to be interviewed, and interviews were completed with 922 respondents (90%), three of whom were subsequently determined to be ineligible. Assuming an 80% eligibility rate for noncontacted households, we estimate that there were 1,663 housing units within the sample frame with an eligible respondent. The overall response rate (number of completed interviews from the number of households in sample estimated to have an eligible respondent) was 55% (919 of 1,663); interviews were completed with 75% of households in which an eligible respondent was identified, and in 90% of the total households in which an eligible respondent was contacted. Sample weights were constructed to adjust for differential selection and response rates, allowing us to estimate population effects from the HEP sample.

For each community member who agreed to participate in the study, data gathered included demographic information (age, income, education); self-reported stressors (life events, police stress, discrimination, safety stress, financial stress); assessments of health-related behaviors; self-reported exposure to airborne PM in home and workplace settings; indicators of social support, integration, and community connectedness; responses to stressful life conditions; self-reported medical history and conditions; anthropomorphic and hemodynamic measures; and nutrition data collected using a food frequency questionnaire. A detailed list of scales used in the survey and supporting documentation are available in Schulz et al. (2005b).

HEP contracted with a survey research organization to manage the day-to-day aspects of the survey and worked closely with this organization to develop and conduct interviewer training and to assist in survey administration. On the basis of recommendations from the HEP SC, survey interviewers were Detroit residents. Members of the HEP SC and other members of their organizations assisted with the 32-hr training in survey interviewing techniques and procedures and instruction in the collection of anthropomorphic and hemodynamic measures. At the completion of training, interviewers received certification and were required to be recertified in collection of survey, anthropomorphic, and hemodynamic measures on a monthly basis. Quality controls included review of completed survey by field supervisors, and additional review of completed surveys by research staff for quality assurance and completeness. The administrator of the subcontracting organization attended monthly meetings of the full HEP SC to provide reports on survey progress and to discuss the quality and progress of the survey.

#### Biomarker data collection.

At the completion of the survey interview, each respondent was invited to participate in the clinical portion of the study, which involved collection of blood and saliva samples. This component of the study allowed for analysis of associations between exposure to social stressors, PM_10_, PM_2.5_, and biomarkers for CVD and, within each focal area, analysis of the potential mediating effects of micronutrients on biomarkers. Of the 919 survey participants, 367 participated in the clinical component of the study, a substantially larger number than the 200 initially anticipated. Each participant was provided with a saliva sample collection kit (Sarstedt Corp., Montreal, Canada) with stepwise instruction for collecting saliva samples adapted from sample collection procedures described in the literature (King et al. 2000). Participants were instructed to collect saliva samples over 2 consecutive days and were asked to store the samples in their home freezer or refrigerator. They were instructed to bring the stored saliva samples to the community site on the day of their scheduled blood draw. Participants were scheduled for their biomarker assessment at a community site (e.g., a community-based partner organization) set up for the purpose of the HEP project in three areas of the city—eastside, northwest, and southwest Detroit. Participants received a reminder phone call from the HEP staff 3 days before their scheduled appointment.

Participants were instructed to fast for 10–12 hr before their appointment and to bring their saliva samples to the site. At the site, their resting blood pressure was measured three times by a team of trained and certified phlebotomists. Participants then completed a brief questionnaire that characterized their use of vitamin, mineral, and herbal supplements, use of prescription and nonprescription medications, and ongoing infection symptomatology. Venous blood was drawn from the participants and aliquoted for processing. Biomarker site staff were trained and required to demonstrate competency and certified in collecting, handling, transporting, and processing of the biomarker samples (Kannan S, Arya I, Schulz A, Wyman L, Roy R, Benjamin A, et al., unpublished data). Training was provided in biohazard safety procedures modified from the Occupational Safety and Environmental Health (OSEH) *Laboratory Biosafety Manual* (OSEH 2005) procedures. Biomarker data collection began in May 2002 and ended in April 2003.

### Follow-up with Results

At the time of data collection, each survey respondent received a card indicating the mean of the second and third measures of blood pressure (systolic, diastolic) taken by the survey interviewer and recommendations for follow-up according to American Heart Association (AHA) guidelines. Reports with results from the food frequency questionnaires and, where relevant, biomarker results were designed by a working group of HEP SC members and computerized by a team of graduate students (Kannan S, Arya I, Benjamin A, Wyman L, Roy R, Schulz A, et al., unpublished data). The dietary reports were produced in Spanish or English, depending on the language in which the survey was conducted, and provided summarized feedback on participants’ dietary intakes based on their responses to the food consumption questionnaire, as well as data on height, weight, and systolic and diastolic blood pressure readings derived from the survey. In addition to feedback about their dietary intakes, blood pressure, height, and weight, suggestions consistent with the AHA nutrition and weight for height recommendations were incorporated within the report. The 367 participants of the biomarker component of the study were provided a second report of their blood pressure (systolic, diastolic) as measured at the biomarker site and blood lipid levels derived from their biomarker site sample. With written permission of participants, in the event that biomarker results indicated elevated risk of CVD based on AHA guidelines, biomarker feedback reports were also mailed to the respondents’ designated health care provider.

Study respondents who indicated an interest received annual mailings with summary results from the study and community outreach and educational activities. Results from HEP data analysis are also disseminated widely through community forums, newsletters, and translation to local decision makers, as well as through peer-reviewed publications. HEP SC members are actively involved in these efforts.

### Data Management and Analysis

#### Neighborhood observation checklist.

NOC data were collected by trained community raters on 551 blocks using a PDA, and data were downloaded electronically to a SAS database (version 8.0, SAS Institute Inc., Cary, NC). IRR across 220 NOC variables was evaluated in two ways. First, we evaluated IRR across the 12 observers, including a gold standard rating on four blocks, using a κ-statistic designed for multiple observers by Gwet (2002) (κ = 0.77). Second, IRR was assessed based on 221 street segments that were rated by two different observers using Cohen’s κ-statistic (κ = 0.77). In addition, test–retest reliability on 54 street segments that the same observer rated when observing adjacent blocks at different time points was high (κ = 0.86). Ecometrics (reliability and validity) for scales created using NOC items were evaluated using processes developed by Raudenbush and Sampson (Raudenbush 2003; Raudenbush and Sampson 1999; Zenk SN, Schulz AJ, Mentz G, House JS, Miranda P, Gravlee CC, et al., unpublished data).

#### Airborne particulate matter.

All filters collected as part of HEP for PM characterization were prepared and analyzed at the University of Michigan Air Quality Laboratory (Keeler et al. 2002; Yip et al. 2004). The detection limit for mass determination, calculated as 3 times the standard deviation of seven replicate filter measures, is 5.1 μg. Upon completion of gravimetric analysis, PM samples collected on Teflon filters were analyzed for trace element composition. Teflon sample filters were wetted with 150 μL of ethanol before extraction in 20 mL of 10% HNO_3_ and sonication for 48 hr in an ultrasonic bath. Samples were then diluted with Milli-Q water to 4% vol/vol solutions before passive acid digestion for 1 month. The extracts were then analyzed for a suite of elements by high-resolution inductively coupled plasma-mass spectrometry (ELEMENT2; Finnigan MAT, Austin, TX) similar to methods previously described (Moore et al. 1996).

#### Community survey.

Survey data were entered into a database by data entry personnel at Automated Resources Management Inc. (Ann Arbor, MI), an independent data management corporation. The food frequency questionnaire was entered into a separate database using a modified version of the Block data analysis software (Block et al. 1986, 1994). Each respondent was identified by a code number, with a key listing that matched code numbers to each survey respondent allowing data collected through various mechanisms (survey, biomarker, NOC, air quality) to be linked for analyses. All data gathered in the face-to-face interviews were entered into a database and linked with data from the NOC, census data, air quality data, and biomarker data to create a comprehensive database. Standardized scales assessing stressors, health-related behaviors, social support, and psychosocial responses to stress were constructed by aggregating individual items into the psychosocial constructs described in the preceding sections. Psychometric properties (Cronbach’s α) were calculated for each scale.

Intakes of micro- and macronutrients were calculated by multiplying the frequency of consumption of each unit of food by the nutrition content of the specified portions on the food frequency questionnaire. Food frequency questionnaires were analyzed for macro- and micronutrients using a modified version of the Block diet analysis program (Block et al. 1986, 1994; Kannan S, Arya I, Benjamin A, Wyman L, Roy R, Schulz A, Dvonch JT, et al., unpublished data). Micronutrient intakes were characterized to determine food group contributions to intakes.

The HEP sample deliberately selected specified race or ethnic groups at higher rates in two of the three neighborhoods in order to obtain large enough sample sizes for race by class comparisons across areas of the city. Furthermore, within each selected household, one person was selected at random from all eligible persons who usually resided in the household. This led to an overrepresentation of respondents from households with fewer eligible persons. Finally, response rates varied across the three neighborhoods, and across different sets of sample blocks within neighborhoods.

Weights were constructed to adjust for these design features. The weights consist of two components: an unequal probability of selection adjustment and a poststratification adjustment. The latter adjustment was designed to make the weighted distribution for each neighborhood resemble the distribution of adults 25 or more years of age obtained in Census 2000 (U.S. Census Bureau 2000). The unequal probability of selection adjustment was computed as the inverse of the probability of selection of each household and person with in the household (probabilities of selection were computed for all units at the time of sample selection and retained for just this purpose). The unequal probability adjusted weights were then further adjusted by a poststratification factor to make the weighted sample look like the Census 2000 population in each neighborhood. This poststratification adjustment provides compensation for differential non-response and noncoverage that arose in the survey. The application of these weights to analyses conducted using the HEP sample allows us to estimate population effects from the HEP sample.

#### Biomarker data.

The validity of all bio-marker measurements was checked through examination of biomarker outliers and external quality control programs, such as routine measurement of biomarkers from phantom samples and lab performance in independent quality maintenance programs such as the Micronutrient Measurement Quality Assurance Programs offered by the National Institutes for Standards and Technology (Gaithersburg, MD) and the Centers for Disease Control and Prevention Lipids Standardization Program (Myers et al. 1994). Blood samples were centrifuged to separate the plasma and serum, which were then stored in a –70°C freezer until further analysis of the samples. Measurements will be made for several biomarker domains of lipids, lipoproteins, lipid peroxidation, and homosyteine metabolite concentrations and for oxidative damage and stress.

#### Integrated data analysis.

As described above, unique identification numbers were used to link data gathered through various components of the study. Census data, administrative data, and data from the NOC were located in separate databases and linked to survey respondents using census block, block group, and tract numbers. Air quality data for each of the three areas of the study were linked for analysis using aerial indicators (northwest, eastside, southwest). Linking of data from various sources allows for analysis across the various sources and levels of data collected for HEP (e.g., contextual and behavioral).

Data analysis for the HEP study will test a series of hypotheses regarding relationships among the components of the conceptual model described in this article (Figure 1). Specifically, the analyses will examine bivariate relationships between the intermediate, proximate, and health outcome variables to establish relationships among these various levels of the model. In addition we will conduct multiple regression analyses to examine independent and cumulative effects of exposures in, for example, the social and the physical environments and to test for interactions among predictor variables. Hierarchical linear modeling techniques will then be used to estimate relationships between indicators of neighborhood built environment (e.g., condition of housing, path characteristics), social environment (e.g., territoriality), psychosocial and behavioral risk factors (e.g., perceived stressors, symptoms of depression, physical activity), and cardiovascular risk factors (e.g., systolic blood pressure), controlling for individual characteristics (e.g., age) derived from survey data. Members of the HEP SC are actively engaged in the data analysis process, in interpretation of findings, as co-authors of peer-reviewed journal articles, as presenters at scientific meetings, and in community forums. In keeping with the community outreach and education plan component of this effort, findings will be disseminated through both peer-reviewed publications and presentations at professional meetings and also through a wide range of local, state, and regional audiences, including community residents and city, state, and regional decision makers. The HEP SC prioritized study findings for dissemination, identified media through which to reach these audiences (e.g., local newspapers, community forums, newsletters), and will participate actively in dissemination of results through these venues.

## Discussion

CVD is a major contributor to morbidity and mortality and varies substantially across racial and ethnic groups as well as by SEP. As a CBPR effort, the HEP brings together representatives from community-based organizations, health service organizations, and academic institutions to collectively investigate the contributions of social and physical environments to racial and socioeconomic inequalities in the risk of CVD. Our goal is to contribute to an understanding of, and to inform efforts to eradicate, these disparities.

HEP emerged from priorities identified by the Detroit URC to examine the contributions of environmental factors to health disparities, and the conceptual model that guides the HEP study builds on previous work conducted by partners involved with the URC. This model integrates prior empirical research, the experience and insights of members of the partnership, conceptual models of race-based residential segregation and health, and a vast literature on CVD. This model guides HEP’s analysis of social and physical environments as intermediate factors contributing to CVD risk, mediating relationships between fundamental factors such as race-based residential segregation and concentrated poverty, and more proximate factors (e.g., physical activity, dietary practices) associated with CVD. Representatives from community-based organizations, health service organizations, and academic institutions have been, and will continue to be, involved in all aspects of HEP, from establishing the priorities for research (the contribution of environmental factors to CVD disparities) to informing the conceptual model, determining the study communities, and development and implementation of the data collection processes.

As the largest contributor to all-cause mortality in the United States as well as to racial disparities in mortality, it is essential to understand the factors that contribute to excess cardiovascular mortality among racial, ethnic, and socioeconomic subgroups of the U.S. population. Members of adversely affected communities join health practitioners and academic researchers in their profound interest in understanding and addressing the pathways and processes through which these disparities are produced and sustained (O’Fallon and Dearry 2002). The wide range of measures of both the physical and social environments and the ethnic diversity of the sample are unique features and major strengths of this study, as is the community-based participatory nature of the process with which it was carried out. The wide range of measures permits comparisons that may provide important insights about relationships among racial or ethnic group status, SEP, social environments, physical environments, and more proximate risk factors for CVD. The community-based participatory process allows community residents and representatives from community-based organizations, health service providers, and academic researchers to pool their skills, resources, and knowledge to extend our understanding of the complex pathways through which local environments influence risk of CVD. Perhaps more important, because these diverse groups engage in the process of developing knowledge about CVD, the capacity to disseminate results widely to local decision makers, health care providers, and community residents, as well as through the scientific literature, is enhanced, along with the potential to facilitate effective interventions and policy changes to reduce racial and socioeconomic disparities in CVD.

## Figures and Tables

**Table 1:** Age-adjusted heart disease mortality rates for non-Hispanic black and non-Hispanic white residents of the United States (1999), Michigan (2000), and Detroit (2002).^a^

	All	Non-Hispanic black	Non-Hispanic white
United States^b^	260.4	336.5^c^	263.5^c^
Michigan^d^	285.3	366.5	275.7
Detroit^c^	401.1	409.1	408.8

a All rates are per 100,000 population. Data from

b U.S. Department of Health and Human Services (2001) and Minimo and Smith (2001);

c MDCH (2004);

d Michigan Department of Community Health (MDCH) (2003).

**Table 2:** Racial and ethnic distribution goals and results (number of respondents) for the Healthy Environments Survey for eastside, northwest, and southwest Detroit.

	Eastside Detroit				Northwest Detroit				Southwest Detroit				Total			
	Goal		Actual		Goal		Actual		Goal		Actual		Goal		Actual	
	AP^a^	BP	AP	BP	AP	BP	AP	BP	AP	BP	AP	BP	AP	BP	AP	BP
Non-Hispanic black	134	133	132	126	67	67	102	60	66	67	49	42	267	267	283	228
Hispanic	0	0	0	2	0	0	1	0	100	100	90	87	100	100	91	89
Non-Hispanic white	0	0	2	0	66	67	63	30	67	66	50	49	133	133	115	79
Other	0	0	1	1	0	0	3	5	0		6	8	0	0	10	14
Subtotal	134	133	135	129	133	134	169	95	233	233	195	186	500	500	499	410
Missing^a^	—		3		—		4		—		3		—		8	
Total	267		267		267		268		466		384		1,000		917	

Abbreviations: AP, above poverty; BP, below poverty.

a Respondents missing data on race and income and therefore uncategorizable.
